# Identification and key management of non-transfusion-dependent thalassaemia patients: not a rare but potentially under-recognised condition

**DOI:** 10.1186/s13023-014-0131-7

**Published:** 2014-09-30

**Authors:** Vip Viprakasit, Paul Tyan, Sarayuth Rodmai, Ali T Taher

**Affiliations:** Department of Pediatrics and Thalassemia Center, Faculty of Medicine, Siriraj Hospital, Mahidol University, Bangkok, 10700 Thailand; Hematology and Oncology, Department of Internal Medicine, American University of Beirut Medical Center, PO Box 11–0236, Riad El Solh 1107 2020 Beirut, Lebanon

**Keywords:** Non-transfusion-dependent thalassaemia, Thalassaemia intermedia, HbE disease, HbH disease, Iron chelation, RBC transfusion, Hydroxyurea

## Abstract

Patients with non-transfusion-dependent thalassaemia (NTDT) have a genetic defect or combination of defects that affect haemoglobin synthesis, but which is not severe enough to require regular blood transfusions. The carrier frequency of NTDT is high (up to 80% in some parts of the world) but the prevalence of symptomatic patients varies with geography and is estimated to be from 1 in 100,000 to 1 in 100. NTDT has a variable presentation that may include mild to severe anaemia, enlarged spleen and/or liver, skeletal deformities, growth retardation, elevated serum ferritin and iron overload. The contributing factors to disease progression are ineffective erythropoiesis and increased haemolysis, which lead to chronic anaemia. The body’s attempts to correct the anaemia result in constantly activated erythropoiesis, leading to marrow expansion and extramedullary haematopoiesis. Diagnosis of NTDT is largely clinical but can be confirmed by genetic sequencing. NTDT must be differentiated from other anaemias including sideroblastic anaemia, paroxysmal nocturnal haemoglobinuria, congenital dyserythropoietic anaemia, myelodysplastic syndromes and iron-deficiency anaemia. Management of NTDT is based on managing symptoms, and includes blood transfusions, hydroxyurea treatment, iron chelation and sometimes splenectomy. Prognosis for well managed patients is good, with most patients living a normal life. Since NTDT is mainly prevalent in sub-tropical regions, patients who present in other parts of the world, in particular the Northern hemisphere, might not been correctly recognised and it can be considered a ‘rare’ condition. It is particularly important to identify and diagnose patients early, thereby preventing complications.

## Model case study

A 9-year old girl of South East Asian origin undergoes routine medical check-up by her local physician. The parents had noted that the child is lethargic and that she reaches her development milestones slightly late. She is short for her age, although not quite below the fifth percentile line of the growth chart. Clinical examination shows mild scleral icterus, tachycardia with regular rhythm, II/VI flow murmur over the left sternal border and mild hepatosplenomegaly. Laboratory test results (normal values) were as follows: serum ferritin, 321 ng/mL (6–24 ng/mL); iron 155 μg/dL (50–120 μg/dL); haemoglobin (Hb), 8.1 g/dL (11–16 g/dL); Chem-10^1^ within normal limits; total bilirubin 3.4 mg/dL (0–1.5 mg/dL). The blood smear shows mild microcytic anaemia (mean corpuscular volume [MCV] 64 fL [70–86 fL], mean corpuscular Hb [MCH] 19.5 pg [28–32 pg], mean corpuscular Hb concentration [MCHC] 28.5 g/dL [11.5–13.5 g/dL], RBC distribution 27.9% [11.5–14.5%]) and reticulocyte count 8%. There is no evidence of inclusion bodies, but the dichlorophenolindophenol (DCIP) precipitate test is positive. Hb electrophoresis test finds HbE 45% with HbF 55%. She is diagnosed with HbE/β-thalassaemia disease and referred to a specialist for long-term management. A haematologist classifies her as having non-transfusion-dependent thalassaemia (NTDT) and recommends intermittent blood transfusions to overcome growth retardation, vigilant observation during periods of infection for the development of acute haemolysis and annual monitoring of body iron levels from age 10 years onwards, with treatment as required.

## An introduction to non-transfusion-dependent thalassaemia

### Definition

Non-transfusion-dependent thalassaemia (NTDT) describes patients who have an inherited genetic defect or combination of defects that affect Hb chain synthesis and, consequently, the oxygen-carrying capacity of red blood cells [1–4]. NTDT is a broad term that covers several thalassaemia disorders, including HbH disease (ORPHA93616), β-thalassaemia intermedia (ORPHA231222), and HbE/β-thalassaemia (ORPHA231249). Even though NTDT patients are often anaemic, they do not rely on blood transfusions for daily function and survival, which differentiates them from patients with thalassaemia major (TM) or transfusion-dependent thalassaemia (TDT). However, abnormal RBC synthesis can cause debilitating clinical symptoms that impact morbidity, quality of life and mortality [4–8]. The transfusion requirements of a NTDT patient can change over time and there is also considerable variability between the transfusion needs of different individuals [7]. The patient described in the case study fits the definition of NTDT, because she is not reliant on blood transfusions for daily function and survival. Nevertheless, the symptoms of growth retardation indicate a need for treatment.

### Genetics and epidemiology of NTDT

Genetic defects that affect the synthesis of globin chains can be divided into two broad groups: 1) a defect(s) in one or more of the genes that code for β-globin chain, 2) a defect(s) in one or more of the genes that code for α-globin chain [5–7]. To date, more than 300 different thalassaemia defects of the globin chains have been discovered and these can be further classified according to their type, *i.e.*, insertion, deletion or base substitution [5–7,9].

β-thalassaemia, which results from one or more defects in the genes that code for the β-globin chain, or α-globin gene rearrangements causing duplication, has 80 to 90 million carriers worldwide (1.5% of the global population) [5]. The highest prevalence is found in India, Bangladesh and South East Asia (where the carrier frequency is approximately 1–5%) [8,10,11]. One β-globin gene defect that is highly prevalent in this population is a single-point mutation (HBB:c.79G > A) causing Haemoglobin E (HbE) [5].

In the past, α- and β-thalassaemias were restricted to malaria-endemic tropical and subtropical regions [6,12,13]. However, in recent years, human global migration from these regions has caused an increase in these conditions in countries previously relatively unaffected by thalassaemias, such as those in North Europe and North America [6,12,13]. Therefore, thalassaemia syndromes are no longer ‘rare’ conditions in such regions and warrant awareness from all health care providers involved. The genotypes and phenotypes of thalassaemia are shown in Table 1.

**Table 1:** **Genotypes and phenotypes of thalassaemia**

	**Variant**	**Genotype**	**Phenotype**
α-thalassaemia	Normal	αα/αα	Normal
	Silent Carrier	-α/αα	Haematologically silent or mild reduction of MCH/MCV
	Minor	-α/-α , - -/αα	Borderline anaemia or normal, as well as microcytic and hypochromic red blood cells
	HbH disease*	**- -/-α , - -/α** ^**T**^ **α, α** ^**T**^ **α/α** ^**T**^ **α**	**Majority have mild to moderate anaemia and marked microcytosis and hypochromia, only few have phenotype similar to thalassaemia major**
	Barts Hydrops Foetalis	- -/- -	Most develop hydrops foetalis syndrome and die *in utero*, or shortly after birth
β-thalassaemia	Normal	β/β	Normal
	Minor	β/β^+^ , β/β^0^	Borderline anaemia or normal as well as microcytic and hypochromic red blood cells
	β-thalassaemia intermedia*	**β** ^**+**^ **/β** ^**+**^ **,β** ^**0**^ **/β** ^**+**^ **, β** ^**+**^ **/β, β** ^**0**^ **/β, β** ^**0**^ **/β** ^**0**^	**Severity is very variable. Clinical picture ranges from mild to moderate NTDT**
	Major	β^0^β^0^ , β^0^/β^+^	Severe anaemia requiring regular transfusions (TDT)
HbE	HbE trait	β^E^/β	Asymptomatic condition with no clinical relevance
	Homozygous HbE	β^E^/β^E^	Usually asymptomatic with borderline asymptomatic anaemia and no haemolysis
	HbE/ β-thalassaemia*	**β** ^**E**^ **/β** ^**+**^ **,β** ^**E**^ **/β** ^**0**^	**Severity is very variable. Clinical picture ranges from NTDT to TDT**
	HbE/HbS	β^E^/β^S^	Similar to sickle cell disease usually with rare vaso-occlusive crisis

*Thalassaemia genotypes that often result in NTDT.

An estimated 300,000 children are born each year with a genetic defect of one or more genes that code for α/β-globin chains [4]. If the defect(s) affect(s) the expression of two corresponding α- or β-globin genes, then phenotypic thalassaemia develops [4].

The clinical course of thalassaemia varies greatly and depends on the combination of genetic anomalies in a patient [14,15]. People with deleterious genetic defects on both alleles coding for β-globins develop the most severe form of β-thalassaemia, namely β-thalassaemia major. The most severe form of α-thalassaemia is hydrops foetalis with Hb Barts, a fatal disorder that results from genetic deletions that abolish the expression of all α genes. Genetic defects other than those resulting in β-thalassaemia major and hydrops foetalis encompass a wide spectrum of severity, from asymptomatic to mild and from moderately severe to severe. The severity of a thalassaemic disorder is associated with the extent of anaemia and consequently the need for therapeutic strategies to overcome anaemia. So, patients with a severe thalassaemia, such as β-thalassaemia major, develop debilitating anaemia and are transfusion dependent. Patients with milder forms of thalassaemia, such as β-thalassaemia intermedia, HbE, and HbH disease develop anaemia, but it is not so severe that they are dependent on RBC transfusions for survival. This group of patients is therefore considered to have NTDT [1–7].

### Pathophysiology of NTDT

**Figure 1:**
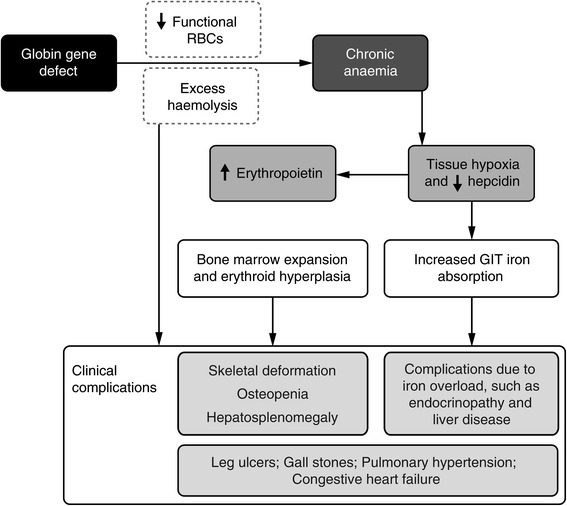
**The pathophysiology of clinical complications of NTDT.**

The main drivers underlying the pathophysiology of NTDT are chronic anaemia; ineffective erythropoiesis and chronic haemolysis from peripheral destruction of RBCs; and compensatory physiological mechanisms that attempt to rectify a state of anaemia, including marrow expansion, extramedullary haematopoiesis and increased absorption of gastrointestinal iron. Because these mechanisms cannot overcome the genetic defect in synthesizing fully functional RBCs, they are constantly activated, leading to the development of diverse clinical complications (see Figure 1) [5–7,16–20]. The ways in which the main pathophysiological drivers of NTDT mediate clinical complications are explained below.

### Chronic anaemia

NTDT patients have underlying anaemia with Hb levels varying between 7–11 g/dL [18,20,21]. Due to imbalances in α- and β-globin chains, globin tetramers are unstable resulting in precipitation and degradation. This process releases free iron and leads to the formation of reactive oxygen species, causing membrane damage and ultimately premature cell death in bone marrow (ineffective erythropoiesis) or peripheral circulation (haemolysis) [22,23]. The severity of anaemia is determined by both the extent of ineffective erythropoiesis and the extent of haemolysis [24]. Haemolysis is associated with progressive splenomegaly and can also contribute to a hypercoagulable state, which many NTDT patients experience. Anaemia can be severe and life-threatening in NTDT patients who experience a physiological challenge, such as illness, trauma or pregnancy; and can also cause growth failure and delayed development [25,26]. The patient described in our case study clearly had growth failure; this is most likely to be a result of chronic anaemia. Her Hb level at the time of presentation was 8.1 g/dL (reference range: 11–16 g/dL).

### Ineffective erythropoiesis

Normal physiological compensation for anaemia is an increase in erythropoietin (EPO) from renal interstitial fibroblasts and hepatic perisinusoidal cells. In thalassaemia, the desired outcome of an increase in functional circulating RBCs is unattainable and EPO stimulation is therefore constantly upregulated [24]. Constant elevation of EPO levels drives erythroid marrow expansion and the development of extramedullary erythroid tissue in the chest, abdomen and pelvis, causing skeletal deformities and osteopenia [27–29]. The inherently defective erythropoiesis in thalassaemia inhibits the differentiation of early erythroid progenitors, resulting in large numbers of these progenitor cells in the liver and spleen. This, together with the excessive amounts of damaged RBCs that are filtered by the spleen, leads to hepatosplenomegaly [24].

### Iron overload

In NTDT, iron uptake from the gastrointestinal tract (GIT) is considerably higher than normal because the upregulated rate of erythropoiesis sequesters large amounts of physiological iron [30]. However, as the sequestered iron is not incorporated into fully functional RBCs, transferrin levels become saturated and unbound physiological iron is deposited in organs, especially the liver. The serum ferritin levels in our case study patient were considerably higher than normal (321 ng/mL compared with the reference range of 30–150 ng/mL), despite her having no history of RBC transfusion. In addition, iron absorption through the gut can be increased due to reduction of hepcidin production in NTDT patients. Hepcidin negatively regulates both duodenal iron absorption and macrophage iron release. Hepcidin binds ferroportin on cells of the intestinal duodenum, macrophages, and cells of the placenta, inducing internalization and degradation. In thalassaemia, overexpression of the erythropoietic factor, growth differentiation factor 15 (GDF15) contributes to iron overload by inhibiting hepcidin expression, and thereby increasing iron absorption through the gut [31,32]. The serum ferritin levels in our case study patient illustrates how extensive iron uptake from the GIT can be in patients with HbE/β-thalassaemia [31,32].

### Other complicating factors

In addition to anaemia, ineffective erythropoiesis and iron overload, several molecular and cellular mechanisms that play a part in the regulation of blood coagulability are dysregulated in NTDT. In these patients, chronic platelet activation, dysregulation of adhesion molecules on vascular endothelial cells and abnormal RBC membranes contribute to a state of hypercoagulability, which increases the risk of thromboembolic complications [33,34].

## Clinical characteristics and common complications of NTDT patients

The clinical characteristics of NTDT patients are associated with the severity of chronic anaemia and all NTDT patients are at risk of developing a wide variety of clinical complications including gallstones, leg ulcers, growth retardation, pulmonary hypertension (PHT), splenomegaly, liver disease and thromboembolic events [16,17,19–21,29,34]. The risks of developing many of these complications are known to increase with age [16,26]. Table 2 lists the most common complications and their prevalence in the different types of thalassaemia associated with NTDT. It is important to note that many patients with NTDT may go undiagnosed, and therefore the frequency of the complications listed are more reflective of those patients with moderate-to-severe NTDT phenotypes.

**Table 2:** Characteristics and complications in NTDT patients* [5,16,20,35–41]

**Characteristics and complications**	**β-thalassaemia intermedia**	**HbE/β-thalassaemia**	**α-thalassaemia syndromes** ^**‡**^
**Presenting age (years)**	Usually >2	Usually >2	Usually >2
**Presenting Hb level (g/dL)**	7–10	9–12 (mild)	8–11
		6 –7 (moderately severe)	
**HbF(%)**	3–50, but can be up to 100	3–50, but can be up to 100	Not raised, but HbH (β_4_) and Hb Barts (γ_4_) present
**HbA2/HbE(%)**	>3.5–4	30–40	<2
**Jaundice**	+	+++	++++
**Growth retardation**	+	+++	+
**Bone and skeletal abnormalities**	+	+++	++++
**Splenomegaly**	++++	++++	++
**Leg ulcers**	++	+	+
**Cholelithiasis**	+++	+	++
**Acute haemolytic episodes**	+	++	+++
**Thrombotic events**	++	+	+
**Extramedullary haematopoiesis**	++	+	+
**PHT**	+++	+	+

*Viprakasit V, unpublished data.

^‡^α-thalassaemia syndromes include deletional HbH and non-deletional HbH disease.

Frequency of complications are expressed as:

0-10%: +

10-30%: ++

30-60%: +++

60-100%: ++++.

### β-thalassaemia intermedia

Patients with β-thalassaemia intermedia usually present when they are ≥2 years of age and their Hb level normally lies between 7–10 g/dL [20]. They are generally considered non-transfusion dependent, but at the more severe end of the spectrum, this may not be the case. Children with Hb levels in the range of 5–6 g/dL often develop skeletal deformities. Patients with Hb levels in the range of 6–9 g/dL develop normally, but they are prone to progressive splenomegaly and declining Hb levels later in life, or when physiological stressors such as infection or pregnancy complicate their condition. β-thalassaemia intermedia patients have an increased susceptibility to infections and skeletal changes and they require individualized treatment strategies because their clinical severity lies between β-thalassaemia major and minor [20]. Management of these patients is particularly challenging for clinicians because they can present with a wide variety of complications which can vary in severity. In addition, until recently there were few clear guidelines on treating the underlying disease (the Thalassaemia International Federation guidelines for the management of NTDT were released recently) [42].

### HbE/β-thalassaemia

This disease can be classified into three categories on the basis of clinical severity: mild (observed in 15% of patients with this disease in South East Asia); moderately severe (represents the majority of HbE/β-thalassaemia cases); and severe. It is estimated that up to half of all patients with HbE/β-thalassaemia exhibit clinical symptoms resembling those of β-thalassaemia major, while the remainder bear a clinical resemblance to the phenotype of β-thalassaemia intermedia or NTDT [35].

### HbH disease

Patients with HbH disease can be classified as having either a non-deletional or a deletional defect of the α-globin genes. Patients with a non-deletional defect generally have lower levels of Hb and higher levels of HbH and inclusion bodies than those who have a deletional defect [36]. Non-deletional HbH is therefore considered a more severe form of the disease than deletional HbH. In addition, patients with non-deletional HbH disease were found to have more symptoms at a younger age, more severe haemolytic anaemia, more growth retardation, more dysmorphic facial features, larger spleens, larger livers, and higher serum ferritin levels [18,36]. These patients required more transfusions than patients with deletional HbH disease [18]. All patients with HbH disease are prone to infections, leg ulcers and gallstones; and those older than 45 years of age are susceptible to iron overload even if they do not receive regular blood transfusions [6,18,36,43]. HbH and α-thalassaemia syndrome has been recently reviewed and hence we will not discuss in detail here [44].

## Making a diagnosis

### Identifying NTDT patients

Many of the clinical complications common in NTDT can be prevented by early intervention strategies. A diagnosis of NTDT before the onset of clinical complications is therefore a valuable investment in the future health of patients. In addition, screening families in high frequency areas may well offer clinical value and will allow for prenatal counselling.

Children with NTDT commonly present with symptoms of anaemia, such as pallor, fatigue, dizziness or vertigo, tachycardia and dyspnoea [36]. Children are usually older than two years of age at the time of presentation and those with mild anaemia can present much later [20]. Our case study patient presented with several symptoms of anaemia. Her parents described her as being lethargic, which is a sign of fatigue, and clinical examination revealed pallor (mild scleral icterus) and tachycardia. In addition, this child was nine years of age at the time of presentation, suggesting NTDT rather than β-thalassaemia major.

NTDT patients with moderately severe phenotype can develop a haemolytic crisis during an acute pathogenic infection or high fever. In a relatively small percentage of these patients, this event triggers investigation into the presence of an underlying haemoglobinopathy. The Hb levels of NTDT patients experiencing a haemolytic crisis rapidly decline and as a result they can be mistaken for having TDT and erroneously entered into a management programme of lifelong, regular transfusions. It is therefore advised that once children recover from the underlying factor that initiated a haemolytic crisis, transfusion therapy be interrupted so that appropriate baseline Hb levels can be determined [45]. In our practices, we will follow such cases with monthly complete blood count (CBC) monitoring for at least 6 months to determine their baseline Hb and associated clinical symptoms such as clinical anaemia, fatigue, lethargy, poor feeding, poor weight gain, intercurrent infection, liver and spleen size and development of bone deformities. These parameters will be carefully considered to categorize patients into either TDT or NTDT phenotypes.

### The diagnostic work-up

**Figure 2:**
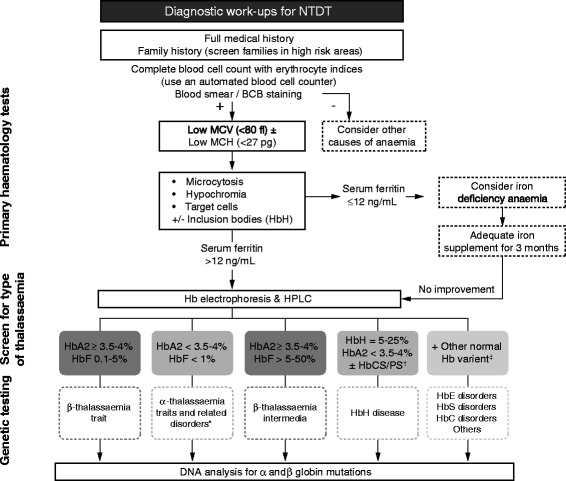
**Diagnostic algorithm for NTDT.** *α-thalassaemia traits and related disorders include α^0^ and α^+^-thalassaemia by deletions and non-deletional α-thalassaemia mutations. ^†^There are two main types of HbH disease: 1) deletional HbH due to deletions (- -/-α) and; 2) non-deletional HbH disease caused by α^0^-thalassaemia and non-deletional mutation (--/α^T^α). ^‡^The common disorders associated with Hb variants include homozygous HbE, HbE/β­thalassaemia and HbE with other variants such as HbE/HbS or HbE/HbC or HbE/HbD, HbS (Sickle), HbS/β-thalassaemia, homozygous HbC and HbC/β­thalassaemia. These diagnoses can be confirmed using appropriate globin genotyping.

The diagnostic protocol for NTDT uses complete blood cell counts with erythrocyte indices as measured by an automated blood cell counter. Typically, blood analyses of almost all thalassaemias show a reduction in the size and Hb content of mature RBCs, which is evident in a reduced MCV and mean cell MCH [46]. Low MCV (<80 fl) and/or MCH (<27 pg) require further investigation by means of electrophoresis, high-pressure liquid chromatography (HPLC) and DNA analysis to identify the thalassaemia type and the genetic defects as shown in Figure 2 [46–48]. If NTDT is suspected, family studies are an important aspect of diagnosis.

There are inexpensive screening tests available, but they utilize the reduced osmotic fragility characteristic of the RBCs of thalassaemia and therefore do not always distinguish thalassaemia from iron deficiency anaemias. A short period (up to 3 months) of iron supplementation (4–6 mg/kg/day of elemental iron) is recommended in cases with unclear diagnosis. If there is no significant improvement (increased Hb, MCV and MCH values), a further determination for NTDT by Hb and DNA analyses should be pursued. Other causes of anaemia such as sideroblastic anaemia, congenital dyserythropoietic anaemia, paroxysmal nocturnal haemoglobinuria, myelodysplastic syndromes and other anaemias including nutritional anaemias such as megaloblastic anaemia should be considered and differentiated from NTDT patients. Patients with β-thalassaemia usually have elevated HbA_2_ (except those with silent β-thalassaemia defects), therefore, combining the results of 1) a test that measures HbA_2_ level and 2) the results of a full blood count (MCH and MCV) is an appropriate initial screening test for β-thalassaemia [4]. Identification of α-thalassaemia is more complicated and the only fail-safe assessment is DNA analysis, though inclusion bodies visible in the blood smear is an important indicator [4,49]. Figure 2 shows an algorithm for diagnosis of α- and β-thalassaemia syndromes associated with NTDT phenotype. However, even though NTDT can be defined by genotype, the diagnosis is mainly clinical and it is based on the severity of the patient’s condition [20]. Table 3 is a guide to differentiate between TDT and NTDT patients.

**Table 3:** **Differentiation between TDT and NTDT**

	**TDT more likely**	**NTDT more likely**
**Clinical**		
Presentation (years)	<2	>2
Hb levels (g/dL)	6–7	8–10
Liver/spleen enlargement	Severe	Moderate to severe
Growth retardation/pubertal failure*	+++/++++	Negative to ++
Clinical anaemia affecting daily living	Yes	No
Bone deformity/thalassaemic facie	Yes	Negative to mild
**Haematologic**		
Nucleated RBC (mm^3^)	Numerous	Negative to few
Reticulocytosis	≥10% of RBC	<10% RBC
**Molecular**		
Type of globin defects	Severe	Mild/silent
Co-inheritance of ameliorating genetic modifiers^†^	No	Yes
Co-inheritance of deteriorating genetic modifiers^‡^	Yes	No

Modified from Thalassaemia International Federation Guideline (2008 second edition).

*Growth retardation; ++, P < 25P, +++, <10P and ++++ = <3P;

^†^Ameliorating genetic modifiers represent genetic factors that can reduce globin imbalance such as α-thalassaemia and/or quantitative trait loci that increase γ-globin expression in *β-thalassaemia intermedia* or *HbE/β-thalassaemia*, *β-thalassaemia* gene in HbH disease.

^‡^Deteriorating genetic modifiers include genetic polymorphisms that can further advance disease severity directly and indirectly such as multiple alpha globin gene rearrangements, genetic haemochromatosis, vitamin D receptor, UGT1A1, α-Hb stabilizing protein polymorphism etc.

## An overview of management strategies in NTDT

Patients with NTDT should be followed by a specialist for long-term management, they should therefore be referred to a haematologist once diagnosed. Long-term therapeutic strategies for NTDT include blood transfusion; hydroxyurea (HU) administration; iron chelation therapy; and splenectomy in cases of severe splenomegaly and/or hypersplenism. Even though specialist haematologists usually manage the long-term therapy of NTDT patients, family physicians should be knowledgeable about the key components of therapy because NTDT patients also seek medical attention for problems that are unrelated to NTDT. The prescription of treatment for medical problems unrelated to NTDT should be done with consideration of the therapeutic strategies that are a part of the long-term management of NTDT.

### Blood transfusion therapy

The initiation of transfusion in NTDT patients can have clinical as well as prophylactic benefits. The OPTIMAL CARE study showed that patients given intermittent or regular transfusions developed fewer clinical complications that are usually linked to chronic anaemia, but they were more prone to develop iron overload-related endocrinopathy [21]. Several observational studies have reported fewer thromboembolic events, PHT and silent brain infarcts in NTDT patients who receive intermittent transfusions versus those who are never transfused [50]. There is also evidence to suggest that the health-related quality of life of NTDT patients is worse than regularly transfused patients [51]. Taher *et al.* have suggested raising baseline Hb for NTDT patients to over 9 g/dL in order to prevent future complications as a prophylactic measure [52]. Nevertheless, it remains important for practicing physicians to communicate and discuss with their NTDT patients in order to tailor their transfusion care to suit the patient’s level of compliance and the resource available in their own healthcare service.

### Hydroxyurea

HU, as an inducer of HbF, has been tested in patients with β-thalassaemia major, β-thalassaemia intermedia and HbE/β-thalassaemia. This compound affects stem cell differentiation in the bone marrow and promotes selective advantages of F-cells and stimulates post-natal expression of γ-globin genes in order to up-regulate HbF production. This results in less globin imbalance and reduced ineffective erythropoiesis. However, increases in Hb after initiation of HU have been shown to vary between 0.6–2.5 g/dL and several NTDT patients did not respond at all to HU [53–55]. Evidence suggests that the response to HU treatment is likely to be dependent on several clinical and genetic factors including Xmn I polymorphism [56,57]. The OPTIMAL CARE study showed that in patients with β-thalassaemia intermedia, HU has clinical benefit, especially when combined with transfusion and iron chelation therapy [21]. Short- and medium-term administration of HU was also well tolerated by β-TI patients, but the effectiveness seems to decline with long-term use [54].

### Iron chelation therapy

It is essential to monitor physiological iron loads in NTDT because of the abnormally high uptake of iron from the GIT in these patients [30,31]. RBC transfusions, given intermittently or on a fairly regular basis, also contribute to systemic iron accumulation.

It is known that NTDT patients develop extensive liver iron loading although their serum ferritin levels are relatively low compared with the serum ferritin levels indicative of liver iron loading in transfusion-dependent patients [58,59]. This complicates the monitoring of physiological iron loads in NTDT, because the current thresholds for serum ferritin used to guide chelation therapy in transfusion-dependent patients cannot be extrapolated to NTDT patients. Taher and Viprakasit *et al.* recently developed a treatment algorithm based on the specific relationship that exists between serum ferritin levels and liver iron concentration in NTDT to guide a decision on chelation initiation in these patients [60]. In NTDT, direct assessment of liver iron concentration, by means of biopsy or magnetic resonance imaging (MRI) analysis every 1–2 years is recommended. MRI analysis has become the mainstay of treatment, as it very accurate and less invasive. In areas where MRI analysis is not readily available, an algorithm that relates serum ferritin to liver iron concentration has been proposed. A decision to initiate chelation therapy should be based on the extent of iron overload and the rate of iron accumulation.

The OPTIMAL CARE study showed that, in NTDT, effective iron chelation can keep serum ferritin levels relatively low, thereby preventing the development of clinical complications due to iron overload [21,61–63]. In addition, NTDT (β-thalassaemia intermedia) patients who received transfusion as well as chelation therapy were shown to have a lower incidence of complications compared with patients who received no treatment or either therapy alone [21]. It is therefore evident that, in many NTDT patients, RBC transfusion given in combination with chelation therapy can have considerable short- and long-term benefits. As chelation therapy can counter organ iron loading, which is an important disadvantage of RBC transfusion, NTDT patients can derive more benefits from transfusion-induced improvement of Hb levels.

### Splenectomy

All NTDT patients, regardless of transfusion pattern, experience an increase in spleen volume over time [16]. This, in turn, causes a worsening of anaemia and consequently, an increased demand for RBC transfusion [1,2,16]. Patients with hypersplenism may also experience leucopoenia and thrombocytopenia, which can lead to recurrent bacterial infection or bleeding. In severe cases, patients develop splenomegaly, which is accompanied by symptoms such as left upper quadrant pain and early satiety. These patients are at risk of developing splenic rupture.

A decision to splenectomize an NTDT patient is complex as there is evidence that in these patients, splenectomy can increase the risk of complications such as thromboembolic events and infection [64]. Results from the OPTIMAL CARE study showed that in patients with NTDT, there is an independent association between splenectomy and an increase in the occurrence of thromboembolism, PHT, heart failure, iron-related endocrinopathy and leg ulcers [21]. Splenectomy is also known to increase the risk of infection, which, in turn, has a high risk for mortality, especially in young patients [65,66]. However, splenectomy with appropriate preventive measures including vaccination and antibiotic prophylaxis is still recommended for patients with HbH disease who almost always respond to this treatment modality and rarely require further blood transfusions [44].

## Early detection of NTDT: the role of the family physician

**Figure 3:**
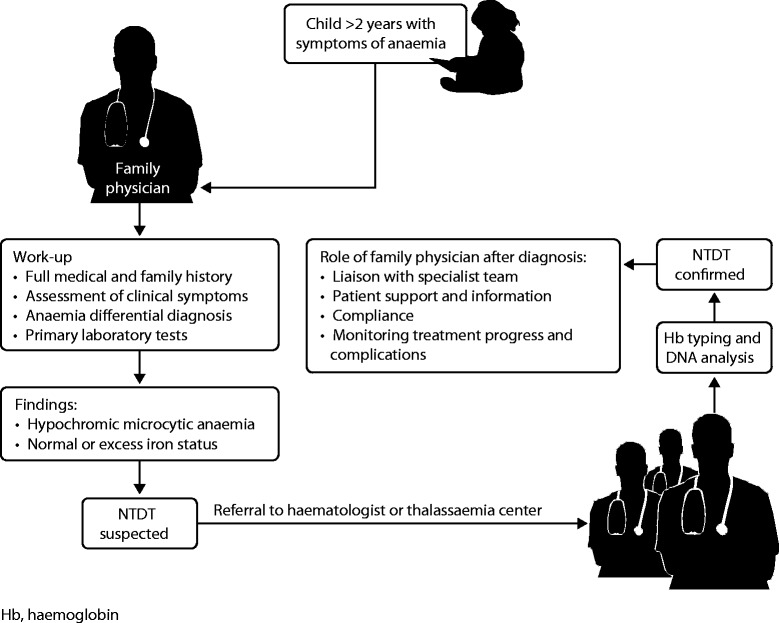
**Algorithm showing the role of the family physician in identifying and managing an NTDT patient.**

An algorithm is presented in Figure 3 to outline the role of the family (or primary) physician in identification and management of NTDT patients. Family physicians are usually the first point-of-contact with patients and therefore an understanding of NTDT presentation will help to facilitate quick diagnosis and appropriate referral. Patients with NTDT require specialist care for long-term management, and should therefore be referred to a haematologist or specialist thalassaemia centre (where available) immediately after diagnosis. Even though the patient’s treatment will be managed by a specialist, awareness of ongoing NTDT therapy is important when prescribing treatment for non-NTDT related medical problems and encouraging patients to remain compliant with their treatment plan. In addition, the patient or family member might come to the family physician for advice and information about NTDT. A healthy lifestyle should be encouraged and patients should avoid smoking, prolonged immobilization and oral contraceptives. For patients with a history of thrombosis, aspirin might be beneficial. Tea consumption should be encouraged in NTDT patients, as it may have some benefit in decreasing iron absorption from the gut [42].

## Conclusion

NTDT is a recently introduced term used to describe thalassaemia phenotypes that do not depend on regular blood transfusions for survival and daily function. The case study described at the beginning of our review illustrates how NTDT patients can present in a family medical practice. Patients with NTDT develop unique combination of clinical complications because they suffer from chronic anaemia, ineffective erythropoiesis and increased iron absorption through the GIT. The recent advances in our understanding of the thalassaemia types and the clinical complications associated with NTDT have made it possible to improve the long-term management of these patients. A timely diagnosis of NTDT can considerably improve outcomes, and all patients who present with anaemia should have appropriate laboratory blood analyses, including full blood counts and blood smears. A diagnosis of microcytic hypochromic anaemia requires further investigation by means of Hb analysis by HPLC or electrophoresis. In addition, DNA analysis is necessary to determine the exact thalassaemia disorder, especially for patients with α-thalassaemia syndromes. Patients with NTDT require specialist care for long-term management. Therapeutic approaches can include intermittent blood transfusion, HU administration and iron chelation therapy.

## Endnote

^1^Chem-10 includes: BUN (Blood urea nitrogen); CA (Calcium); Na (Sodium); K (Potassium); Cl (Chloride); CO2 (Bicarbonate); Creatinine; Glucose; Phosphorus; GGT (Gamma-glutamyl transpeptidase).

## Abbreviations

CBC: Complete blood count
CS: Constant spring
EPO: Erythropoietin
GIT: Gastrointestinal tract
Hb: Haemoglobin
HbPS: Hb Paksé
HPLC: High-pressure liquid chromatography
HU: Hydroxyurea
MCH: Mean corpuscular haemoglobin
MCHC: Mean corpuscular haemoglobin concentration
MCV: Mean corpuscular volume
MRI: Magnetic resonance imaging
NTDT: Non-transfusion dependent thalassaemia
PHT: Pulmonary hypertension
RBC: Red blood cell
TDT: Transfusion-dependent thalassaemia
TI: Beta-thalassaemia intermedia
TM: Thalassaemia major
